# A Highly Stretchable Force Sensitive and Temperature Sensitive Sensor Material with the Sandwich Structure of PDMS + PDMS/GaInSn + PDMS

**DOI:** 10.3390/polym15183776

**Published:** 2023-09-15

**Authors:** Rongmin Zhang, Qianqian Zhai, Fandou Bao, Di Zhao, Zhihua Lu, Jing Wang, Weina Wang

**Affiliations:** School of Physical Science and Intelligent Engineering, Jining University, Qufu 273155, China; zrongm@163.com (R.Z.);

**Keywords:** flexible conductive sensor material, sandwich structure, force sensitive, temperature sensitive, polydimethylsiloxane, GaInSn

## Abstract

Flexible conductive sensor materials have received great attention for their sensitive electrical response to external conditions and their promising applications in flexible wearable and robotic applications. In this work, a highly stretchable force sensitive and temperature sensitive sensor material with a sandwich structure was prepared from the polydimethylsiloxane (PDMS) and the liquid metal (LM) gallium–indium–tin alloy (GaInSn). The sandwich structure (PDMS + PDMS/GaInSn + PDMS) was proven to prevent the “leakage” of LM. The preparation method of the sensing material was simple and time-saving (less than 1.5 h) and can be used for industrial production. The electrical performance analysis results confirmed that the resistance (*R*) of the material was sensitive to the external force, such as repeated stretching, compressing, bending, and impacting. The Δ*R*/*R* changed periodically and stably with the repeated stretching, when the GaInSn/Part A ≥ 0.4, the cyclic tensile strain ≤ 50%, and the cyclic tensile rate ≤ 2.5 mm/min. The *R* of the sensor materials was also responsive to the temperature, such as hot air and liquid nitrogen. In conclusion, this work provides a method for preparing sensing materials with the sandwich structure, which was confirmed to be sensitive to force and temperature without leaking LM, and it produced different types of *R* signals under different deformations and different temperatures.

## 1. Introduction

Flexible electronics technology is a technology that builds electronic devices and forms circuits on a flexible substrate. It can ensure that its conductive properties are not affected under deformation conditions or changes regularly with deformation, and it has a huge application prospect in sensing, energy storage, electronic medicine, human– computer interaction, and other aspects [1]. Flexible wearable devices, in particular, can convert external stimuli such as pressure and temperature into electrical signals, and their applications in sensors and electronic skin have attracted wide attention [2]. From the perspective of materials, the development of high-performance flexible conductive materials is the basis of realizing intelligent, flexible, and highly integrated electronic devices. The combination of polymer material and the conductive medium can not only obtain the unique machinability and mechanical properties of the polymer, but also has the electric and thermal conductivity characteristics of the conductive medium [3,4,5].

First, the selection of the conductive medium. The high elastic polymer matrix has been doped with different forms of conductive media such as Ag nanowires [6], Cu nanowires [7], a carbon nanotube conductive scaffold [8], a carbon nanotube “forest” [9], a carbon nanotube coating [10,11], a graphene conductive network [12], graphene nanoplates [13], Mxene [14], etc. The resulting composite material has good ductility, sensitivity, and reproducibility, achieving the dual goals of stretchy and conductive properties. It is mainly used in a retractable battery, strain sensor, electronic skin, and artificial muscle. However, the above conductive media are rigid materials, whose deformation ability does not match that of the flexible matrix materials. In the process of repeated deformation, the two materials are prone to irreversible relative sliding, forming internal stress at the interface joint and causing internal defects [15,16].

Liquid metal (LM) is a kind of metal or alloy with a low melting point, which can form liquid eutectic at room temperature. It has the properties of both fluid and metal and is the best combination of deformability and electrical conductivity among existing materials [1,17]. As a functional flow filler, combined with flexible polymer functional materials, it is a potential flexible electronic device. In recent years, the combination of LM and polymer materials has received extensive attention in the field of flexible electronics [16,18,19,20,21,22,23].

Secondly, the selection of the polymer matrix material. There are various kinds of polymer matrix materials that can be selected when combined with LM, but considering the purpose of flexibility and bionics, polydimethylsiloxane (PDMS) is the most popular choice [15,24,25,26,27,28,29,30,31]. PDMS has an excellent resistance to high and low temperatures and air permeability, good chemical stability, low surface tension, and an elastic modulus close to human skin [27], which makes it an ideal substrate material for the preparation of flexible wearable devices.

Thirdly, the aspect of the compound method. It has been reported that microchannels are prepared on the polymer matrix in advance via lithography and other methods, and LM is injected into the microchannels to prepare the resistance and capacitance sensors [32,33,34,35]. However, this method has a high cost and is only based on the deformation of the microchannels, which limits the mechanical properties and electrical induction ability of materials [16]. Moreover, due to the electromigration (the LM migrates toward the cathode and depletes the anode when undergoing direct current stressing) of LM, it is difficult to apply this method to the conductive circuits [2,36]. In this article, a molding method that mixes LM with viscous polymer materials before curing are reported, and it has been verified to be simple and effective, since it is inexpensive and does not require the fabrication of micropatterns of the matrixes compared to the microchannel method [16].

However, the distribution of the LM microdroplets in the matrix is uncontrollable, and they are easy to be deposited at the bottom of the matrix material during the curing process. In addition, the problems of the LM “leakage” and the leaky LM affecting the surface properties of the PDMS are inevitable. The “sandwich” structure has been verified to be an excellent structure for the sensor application [37]. So, in order to solve the above problems, a sandwich composite structure of PDMS (top) + PDMS/LM (middle) + PDMS (bottom)) was prepared via the method of curing layer by layer under the influence of gravity, which can not only solves the problem of the “leakage” of the LM, but also ensures that the LM will not affect the surface characteristics of the PDMS.

In addition to systematically exploring the prepare method of the flexible and stretchable conductive material with the “sandwich” structure, the force sensitive and temperature sensitive sensor based on this material as the core were also explored, which will have a certain guiding significance for the development of new flexible sensor materials and their potential applications.

## 2. Materials and Methods

### 2.1. Materials

Anhydrous ethanol (AR, ≥99.7%) was obtained from Sinopharm Chemical Reagent Co., Ltd. (Shanghai, China). The gallium–indium–tin alloy (GaInSn, 99.999%, melting point 11 °C) was obtained from Dongguan Dingguan Metal Technology Co., Ltd. (Dongguan, China). PDMS (SYLGARD^TM^ 184 Silicone Elastomer Kit: Part A is mainly polydimethyl-methylvinylsiloxane with tiny amounts of platinum catalysts; Part B is mainly the crosslinking agent polydimethyl-methylhydrogensiloxane; A and B are used with a mass ratio of 10:1), was purchased from DOW SILICONES CORPORATION (Auburn, MI, USA). Copper foil (0.04 mm) was obtained from Nanjing Houzhu Technology Development Co., Ltd. (Nanjing, China). Medical liquid nitrogen (99.9%) was obtained from Shenzhen Founding Alliance Industrial Co., Ltd. (Shenzhen, China). All the reagents above were used without additional treatment.

### 2.2. Preparation of the PDMS + PDMS/GaInSn + PDMS Sensor Material with the Sandwich Structure

**Figure 1:**
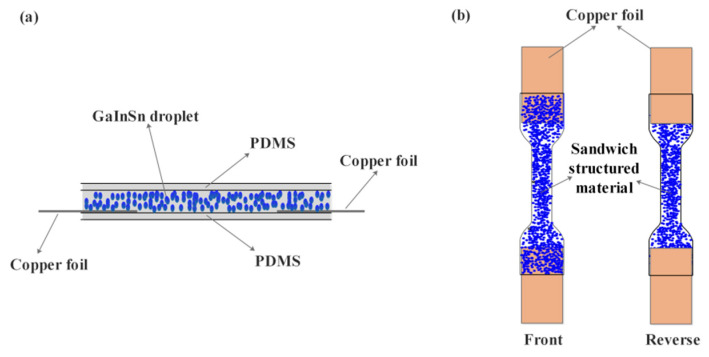
(**a**) Cross section, (**b**) front, and back diagram of the sandwich-structured material.

The PDMS + PDMS/GaInSn + PDMS sensor material with the sandwich structure was prepared via a layer-by-layer curing method under the influence of gravity. First, a certain amount of viscous PDMS (Part A:Part B = 10:1) was poured (the thickness of PDMS is 0.3 mm) into the dumb-bell groove with depths of 2 mm and cured at 100 °C for 0.5 h. An additional reaction occurred between the vinyl of Part A and the Si-H of Part B. Second, two rectangular copper foils were pasted on the terminals of the first layer in the dumb-bell groove. Third, Part A and the GaInSn (Table 1, No. 1~5, GaInSn/Part A = 0, 0.2, 0.4, 0.6, 0.8) were mixed together physically and stirred manually using a glass stick adequately for about 3 min, until the liquid metal GaInSn droplets were no longer visible and the mixture was gray and homogeneous. Then, Part B was added into the mixture and poured onto the first pure PDMS layer, until the dumb-bell groove was filled up, and then was also cured at 100 °C for 0.5 h. The GaInSn droplets distributed in the bottom of the second layer because of gravity; meanwhile, the top of the second layer almost contained no GaInSn droplets. As a result, the PDMS + PDMS/GaInSn + PDMS with the sandwich structure was obtained and emoulded, and the two rectangular copper foils were stuck between the first PDMS layer and the PDMS/GaInSn layer at the two ends of the dumb-bell material, where the GaInSn droplets settled on the copper foils, forming the electric channel. As a result, the sandwich-structured material could be tested without being pierced. The cross section, front, and back diagram of the sandwich-structured material are shown in Figure 1.

In addition, a double-layer structure (as a comparison) was also prepared. The mixture of Part A, GaInSn, and Part B was poured into the dumb-bell groove and cured. Also, the GaInSn droplets settled at the bottom of the material because of gravity. So, a double-layer structure PDMS(top)+PDMS/GaInSn(bottom) was obtained.

### 2.3. Characteristic of the Sandwich-Structured PDMS + PDMS/GaInSn + PDMS Sensor Material

#### 2.3.1. Morphology

The distribution of the LM droplets in the PDMS was observed via the Optical microscope (RH-2000, HIROX, Shanghai Haoshi Instrument Technology Co., Ltd., Shanghai, China).

#### 2.3.2. Thermal Performance

The thermal properties of the five samples with different GaInSn contents were studied via the thermogravimetry calorimeter (TG, Beijing Henven Scientific Instument Factory, HCT-1, Beijing, China), where the samples were heated from room temperature to 800 °C at a rate of 10 °C/min in an atmosphere of N_2_. The effect of GaInSn content on the heat resistance of the sensor materials was discussed.

#### 2.3.3. Deformation and Electrical Signal Measurement

The evolution of the electrical signal-resistance changing (Δ*R*/*R*) with several deformation modes of the sensor material, such as repeated stretching, compressing, bending, and impacting, were measured. The repeated stretching cycle operations and compressing were conducted on an electronic universal testing machine (UTM4104, Shenzhen Suns Technology Stock Co., Ltd., Shenzhen, China). The bending was conducted by binding the samples to a flexional finger model. The impacting was realized when applying a 33 g metal ball suspended on the wall using a 1 m long rope. The Δ*R*/*R* changing with time during the deformation processes were monitored by the Keysight 34461A on a two-wire mode at room temperature. Automatic mode was used with the measurement option set to Resistance 2 W. The strain gauge factor (*GF* = (Δ*R*/*R*)/*ε*, *ε* is the strain) [38,39] was used to represent the sensitivity of the sample during stretching.

#### 2.3.4. Temperature Change and Electrical Signal Measurement

The evolution of the Δ*R*/*R* with temperature was measured. The high and low temperature were realized via a heating blower and a liquid nitrogen spray. The Δ*R*/*R* changing with time during the temperature change processes was also monitored via the Keysight 34461A.

## 3. Results and Discussion

### 3.1. Morphology Analysis

**Figure 2:**
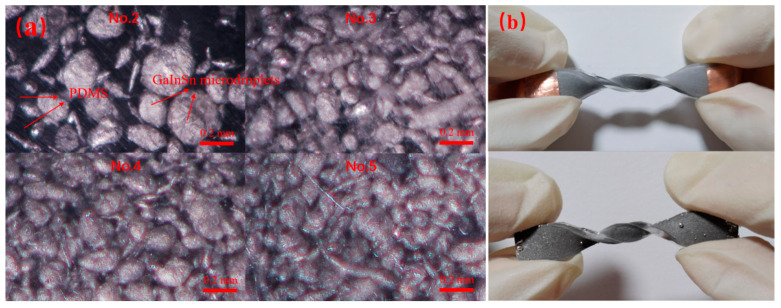
(**a**) The microcosmic distribution of the GaInSn microdroplets in PDMS via optical microscope. (**b**) Comparison of GaInSn “leakage” between the double-layer and the three-layer structure.

The optical microscope pictures of samples No. 2–5 are shown in Figure 2a. The GaInSn microdroplets were evenly distributed in PDMS. The light area was the GaInSn microdroplets, and the dark area was PDMS. It could be seen that with the increase in GaInSn content, the amount of GaInSn microdroplets in the PDMS gradually increased, and the GaInSn microdroplets’ size decreased, which was because the higher the GaInSn content, the more likely it was to collide with the stirring rod during stirring, and the microdroplets became smaller. The GaInSn microdroplets were uniformly dispersed in the PDMS and existed in an irregular spherical and round bar shape. The GaInSn microdroplets were connected with each other, and the higher the content of GaInSn microdroplets, the distance between any two metal droplets decreased. It showed that the GaInSn microdroplets had good dispersion in the PDMS matrix and can form a uniform conductive network. The sandwich structure ensured that the composite material will not cause the LM microdroplets’ “leakage” during the deformations, as shown in Figure 2b, which showed the comparison of the GaInSn “leakage” between the double-layer structure and the three-layer structure (sandwich structure), where it could be seen that the “leakage” of the double-layer structure was serious when twisted 360°, while the sandwich structure had no “leakage” phenomenon.

### 3.2. Thermogravimetry Analysis

**Figure 3:**
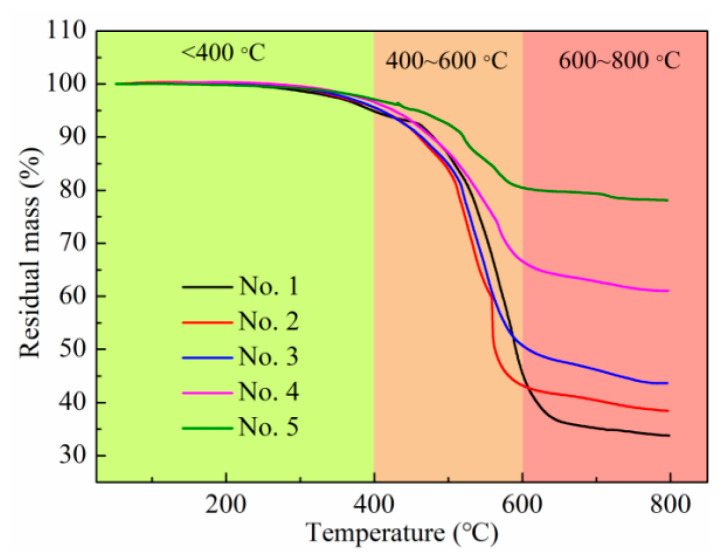
TG curves of the PDMS + PDMS/GaInSn + PDMS samples.

The TG curves of the samples are shown in Figure 3. It could be found that each sample had three stages of weightlessness with the increase in temperature: the stage of slow weightlessness, rapid weightlessness, and stable weightlessness. From room temperature to 400 °C, the slow weight loss of all samples were less than 5%, mainly due to the volatilization of the small molecule compounds, indicating that the composite material had good thermal stability in this temperature range. The weight of each sample began to exhibit loss obviously at 400 °C to 600 °C, and the weight loss rate reached the maximum at about 500 °C, due to the Si-CH_3_ in the composite structure which underwent great thermal degradation. After that, the weight loss rates were gentle. When the temperature reached 600 °C, the high-temperature resistance of the Si~O skeleton made the material no longer decompose, and the qualities of the samples were basically unchanged. The final weight residues of samples 1~5 were 33.8%, 38.4%, 43.6%, 61.1%, and 78.1%, respectively, which was consistent with the GaInSn (boiling point of 1300 °C) contents of the samples, indicating that the thermal weight loss of the composite material was reduced by the addition of the GaInSn.

### 3.3. Deformation Generates Electrical Signal

Firstly, the resistance of each sample was measured via the Keysight 34461A, where the resistance value of the composite material is [40], as shown below:

(1) $$R=\sum_{i=1}^{N}R_{i}+\sum_{i=1}^{N}fR_{c}+\sum_{i=1}^{N}fR_{t}$$

where *R_i_* is the intrinsic resistance of the conductive nanomaterial, *R_c_* is the contact resistance, and *R_t_* is the tunnelling/hopping resistance. The intrinsic resistance of conductive nanomaterials is related to their intrinsic properties. The contact resistance is formed via the interaction of conductive particles, while the tunnelling/hopping resistance is related to the transfer of electrons from one particle to another [41]. When the composite is deformed, it may cause changes in the contact resistance and the tunnelling/hopping resistance. Therefore, deformation of the material can be judged by the change in the resistance.

**Figure 4:**
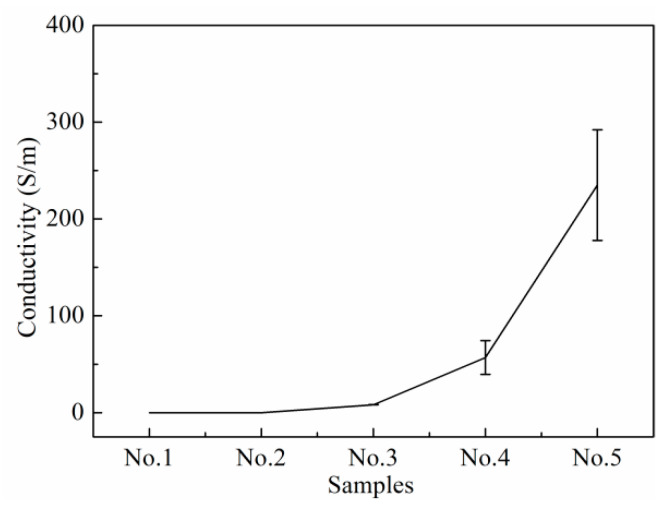
Conductivity of the samples.

The calculated conductivity of the sample is shown in Figure 4. The conductivity of the sample increased with the increase in the GaInSn microdroplets’ content, as the higher the content of GaInSn microdroplets, the closer the combination between them (Figure 2a) and the less contact resistance and tunnelling/hopping resistance. Samples No. 1 and 2 could not form a conductive network, for the GaInSn microdroplets failed to connect with each other because of their low content, as shown in Figure 2a. The samples with GaInSn/Part A above 0.4 could form a conductive network. Sample No. 4 had high conductivity and good repeatability.

**Figure 5:**
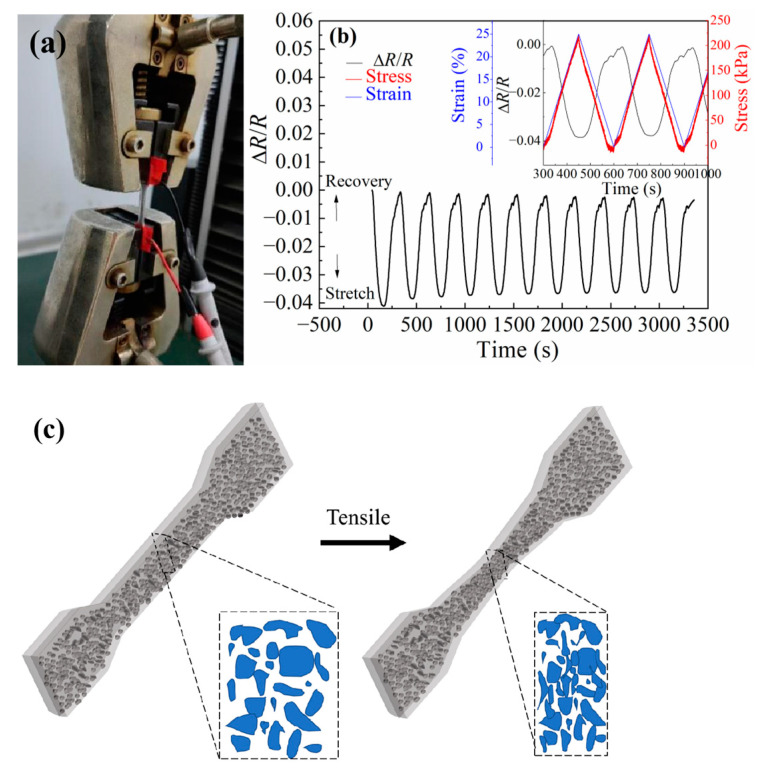
(**a**) Tensile coupling electrical test photograph of PDMS + PDMS/GaInSn + PDMS samples. (**b**) The Δ*R*/*R*, the strain, and the stress evolution of the samples under repeated stretching cycle operations. (**c**) Schematic diagram of conductive network changes during sample stretching.

The electrical signal Δ*R*/*R* were monitored when the samples were stretched repeatedly via the electronic universal testing machine, as shown in Figure 5a. The stress–strain and the Δ*R*/*R* change are shown in Figure 5b. The Δ*R*/*R* of the samples decreased when it was stretched and increased when it was recovery because the samples produced certain strain under the action of tensile stress, including longitudinal elongation and transverse contraction. Although the path in the conductive direction was lengthened, more GaInSn microdroplets contacted with each other to form a smoother conductive path (as shown in Figure 5c), which reduced the total resistance of the samples. On the contrary, when the material recovered, the Δ*R*/*R* also recovered to the initial value when the material was not stretched. There was a good correlation between the stress, the strain, and the electrical signal Δ*R*/*R*. And, there was minimal delay between the Δ*R*/*R* and strain. Therefore, the material could convert the repeated stretching into a stable and cyclic electrical signal, and the material was not damaged during the stretching process.

**Figure 6:**
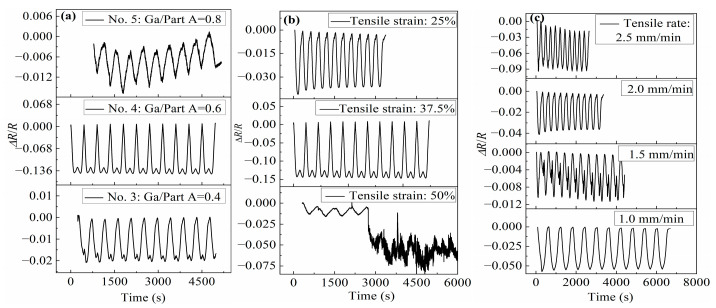
The Δ*R*/*R* evolution of the PDMS + PDMS/GaInSn + PDMS samples under repeated stretching cycle operations: (**a**) different samples, (**b**) different tensile strain, and (**c**) different tensile rate.

Samples No. 3–5 were subjected to a repeated stretching cycle operation, and the stretching and the Δ*R*/*R* testing curves are shown in Figure 6. With a strain of 37.5% at 2 mm/min, as shown in Figure 6a, sample No. 4 was found to have a more stable electrical signal during cyclic stretching and has the highest sensitivity (*GF* = 0.36) among each sample. Therefore, sample No. 4 was chosen as the research object in the following tests.

Sample No. 4 was subjected to a repeated stretching cycle operation at a speed of 2 mm/min and different tensile strains, and its Δ*R*/*R* was measured as shown in Figure 6b. The Δ*R*/*R* of the sample changed periodically with the strain when the tensile strains was 25% and 37.5%. When the tensile strain was 50%, the Δ*R*/*R* varies periodically with the strain during the first few stretching cycles. After several stretching cycles, the resistance dropped sharply. Although the resistance rate still changed periodically with the strain, the resistance value became less stable, indicating that the conductive network formed by the GaInSn microdroplets in the PDMS + PDMS/GaInSn + PDMS sensor material was destroyed by the large deformation. 

Sample No. 4 was also subjected to a repeated stretching cycle operation with a tensile strain of 25% and different tensile rates. The Δ*R*/*R* is shown in Figure 6c. The Δ*R*/*R* changed steadily and periodically with the strain with tensile rates from 1.0 mm/min to 2.5 mm/min. The maximum value of *GF* was 0.36, with a tensile speed of 2.5 mm/min and a strain of 25%, indicating that the sensor material had the highest sensitivity under this condition.

In conclusion, in the repeated stretching cycle operation of the PDMS + PDMS/GaInSn + PDMS sensor material, the Δ*R*/*R* could change periodically and stably with the strain, when the GaInSn/Part A was higher than 0.4, the cyclic tensile strain was less than 50%, and the cyclic tensile rate was no more than 2.5 mm/min.

**Figure 7:**
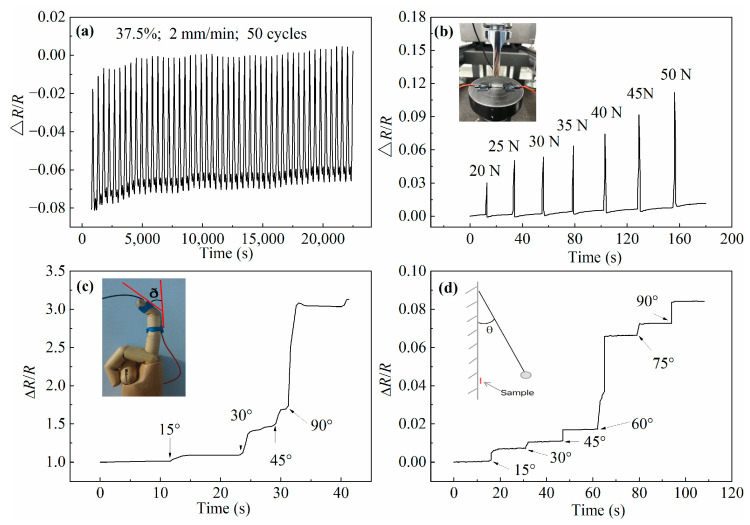
The Δ*R*/*R* evolution of the PDMS + PDMS/GaInSn + PDMS materials resulted from different deformation forms: (**a**) 50 times repeated stretching cycle, (**b**) compressing with different forces, (**c**) bending with different angles, and (**d**) impacting with different swing angles.

In addition to stretching being able to produce electrical signal changes, the influence of other deformation methods, such as compressing, bending, and impacting, on the electrical signal were also studied. The Δ*R*/*R* evolution of the PDMS + PDMS/GaInSn + PDMS sensor materials resulting from different deformation forms are shown in Figure 7. The sample was subjected to cyclic stretching with a tensile strain of 25% and tensile rate of 2 mm/min for 50 cycles and 7 h, as shown in Figure 7a. It could be found that the synthesized PDMS + PDMS/GaInSn + PDMS sensor material had a stable periodic resistance change rate in long working hours.

The Δ*R*/*R* of the material under pressure was studied. The PDMS + PDMS/GaInSn + PDMS sample was fixed and compressed, as shown in the small picture in Figure 7b. The force applied to the sample gradually increased, resulting in an increase in the Δ*R*/*R* value, as shown in the line graph in Figure 7b. The reason was that the pressure on the surface of the sample, resulting in the increase in the distance between the LM droplets in the compressed part of the samples, leads to an increase in the contact resistance and the tunnelling/hopping resistance. The greater the pressure, the more the resistance increased. However, the Δ*R*/*R* returned to 0 after the compressing, proving that the damage to the sample caused by the compressing was temporary and recoverable. 

Then, in order to research the effect of bending on the electrical signals, the sample was tied to the finger model, as shown in the small picture in Figure 7c, and the measured Δ*R*/*R* versus the bending angles(ð) is shown in the line graph in Figure 7c. the finger model was bent at 15°, 30°, 45°, and 90°, where the resistivity of the sample increased obviously and immediately after the bend angle increased. While the bending angle remained constant, the resistance remained stable. The reason was that the side far from the finger of the sample was more deformed than the side near the finger of the sample, and the uneven deformation of the material led to the destruction of the internal conductive network, which resulted in the increase in the resistance. When the sample was bent at 90°, the resistance increased most, indicating that the maximum separation occurs between the LM microdroplets at this stage.

In order to research the effect of the impact process on the electrical signals, the sample and a swing ball were tied to the wall, and the ball was pulled up and released, hitting the sample exactly as shown in the small picture in Figure 7d, and the measured Δ*R*/*R* versus the swing angles is shown in the line graph in Figure 7d. The angle between the cycloid and the wall surface was call the swing angle *θ* (as shown in the small picture in Figure 7d), and the tested electrical signals at *θ* = 15°, 30°, 45°, 60°, 75°, and 90°. As the swing angle increased, the resistance also increased step by step. The larger the swing angle was, the faster the impact speed of the ball against the sample and the larger the destructive energy was. After every impact process, the resistance stayed constant but could not go back to its original value, proving that the impact by the swing ball led to the permanent damage of the conductive network. When the swing angle was 60°, the resistance increased the most, which indicated that the internal conductive network of the sample was damaged the most.

### 3.4. Temperature Change Generates Electrical Signal

**Figure 8:**
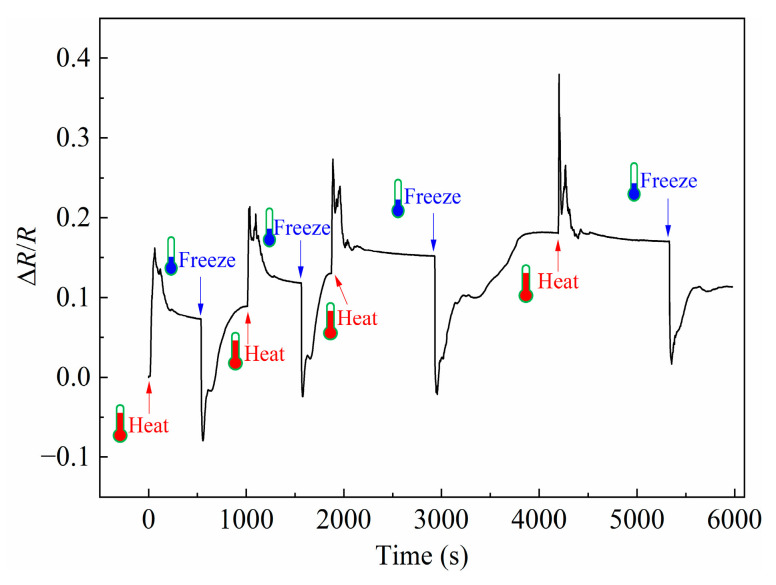
The *ΔR*/*R* evolution of the PDMS + PDMS/GaInSn + PDMS sensor material between the heat and freeze.

The temperature of the PDMS + PDMS/GaInSn + PDMS sensor material was changed, and its Δ*R*/*R* evolution over time was recorded as shown in Figure 8. The Δ*R*/*R* increased when the samples were heated via a heating blower and decreased when frozen via a liquid nitrogen spray. The reason was that the thermal expansion coefficient of PDMS was larger than that of the liquid metal GaInSn. Therefore, when the sample was heated, the distance between the LM microdroplets was widened, and the conductive network was stretched to increase the resistivity. While in the process of freezing, the shrinkage of PDMS was larger than that of the LM GaInSn, and the distance between the LM microdroplets decreased, which made the conductive network shrink and the resistivity decreased. Every time the sample was heated or cooled, the internal conductive network was partially destroyed, resulting in an increase in the resistance baseline.

## 4. Conclusions

A highly stretchable force-sensitive and temperature-sensitive sensor material with a sandwich structure was successfully prepared via the PDMS and the liquid metal GaInSn. The sandwich structure (PDMS + PDMS/GaInSn + PDMS) has been proven to prevent the “leakage” of the LM. The preparation method of this sensing material is simple and time-saving (less than 1.5 h) and can be used for industrial production. The thermal analysis results showed that the addition of liquid metal improves the thermal stability of the sensor material. The electrical performance analysis results confirmed that the resistivity of the material was sensitive to the external force, such as repeated stretching, compressing, bending, and impacting. The Δ*R*/*R* changed periodically and stably with the repeated strain, when the GaInSn/Part A was higher than 0.4, the cyclic tensile strain was less than 50%, and the cyclic tensile rate was no more than 2.5 mm/min. The Δ*R*/*R* of the sensor materials was also responsive to the temperature. In addition, the flexible sensor material produced different types of resistivity signals under different deformations and different temperatures, based on which it can accurately judge the different external environment changes. So, the material can be used as a deformation and temperature sensor in the field of flexible wearable and robotic applications. 

## Figures and Tables

**Table 1:** The raw materials’ consumption of the samples.

Samples	GaInSn/Part A	Part A (g)	Part B (g)	GaInSn (g)
No. 1	0	2.0	0.2	0
No. 2	0.2	2.0	0.2	0.4
No. 3	0.4	2.0	0.2	0.8
No. 4	0.6	2.0	0.2	1.2
No. 5	0.8	2.0	0.2	1.6

## Data Availability

The data that support the findings of this study are available from the corresponding author upon reasonable request.
